# Self-care practices among professional nurses employed in primary health care clinics

**DOI:** 10.4102/safp.v66i1.5955

**Published:** 2024-09-25

**Authors:** George J. Nkabinde-Thamae, Charlene Downing

**Affiliations:** 1 Department of Nursing, Faculty of Health Sciences, University of Johannesburg, Johannesburg, South Africa

**Keywords:** caring, compassion fatigue, practices, professional nurses, self-care

## Abstract

**Background:**

Professional nurses typically promote and advocate self-care practices to their patients to achieve better health outcomes, but rarely engage in these practices themselves.

**Methods:**

A qualitative, descriptive phenomenological approach was used in this study. Ten professional nurses employed in different primary health care facilities were purposively sampled. Semi-structured interviews were conducted to collect data, which were analysed using Colaizzi’s data analysis strategy, and data saturation was reached.

**Results:**

Two themes were identified. Theme 1: the participants expressed that internal and external factors compromised self-care practices, such as subconscious self-neglect, insufficient resources, and a depressed economy, which encouraged them to work extended hours. Theme 2: participants’ holistic well-being was compromised, as they neglected their mental well-being.

**Conclusion:**

Self-care practices among professional nurses seem unachievable. The extent of this population’s self-care neglect was evident during the coronavirus disease 2019 (COVID-19) pandemic when professional nurses globally gave of themselves relentlessly.

**Contribution:**

This is the first study conducted on the topic in the City of Ekurhuleni, and the findings will provide relevant stakeholders with a directive on what strategies, policies, and guidelines to develop and implement to make self-care practices attainable for professional nurses.

## Introduction

Self-care can be defined as the capacity of individuals, families, and communities to enhance their well-being, prevent illnesses, sustain a healthy state, and manage the challenges of sickness and impairment independently or with a healthcare professional’s assistance.^1^ Thus, self-care practices comprise activities that promote an individual’s physical, psychological, social, emotional, and spiritual health and well-being.^2^ The concept of self-care among professional nurses appears to be a known phenomenon. However, since the onset of coronavirus disease 2019 (COVID-19), there has been a surge of studies focusing on professional nurses’ well-being, creating increased awareness of how professional nurses globally struggle with self-care practices.^3^ The pandemic exposed professional nurses to excessive workloads, which resulted in their ill health and inability to provide quality patient care. It also prevented professional nurses from preserving their health and well-being through self-care practices.^4^ A systematic and integrative review revealed that professional nurses and nurse students focus on promoting patients’ self-care practices and often pay minimal attention to or neglect their own.^5^ Throughout the nursing profession, it has been observed that professional nurses prioritise their patients and others.^6^ As a manager in a primary health care setting, the primary author has observed that professional nurses struggle to practise self-care. These anecdotal experiences were confirmed in a systematic review that found professional nurses prioritise care for their patients and family members to their detriment. Being endlessly available and selfless can lead to burnout and compassion fatigue.^7^

Self-care practices should be part of professional nurses’ daily routine to maintain their health needs and reserve their energy to help others.^8^ The nursing profession has been labelled as a high-expectation and risky occupation, which illustrates the need for professional nurses to be mindful that self-care practices are not selfish but a way of being emotionally intelligent.^9^ Professional nurses are exposed to various challenges, such as increased patient workloads, workplace challenges, infections, burnout, and compassion fatigue, which make self-care practices almost impossible in their professional and personal lives.^10,11^ Despite the known challenges within the nursing profession, compassion fatigue appears to be the main contributing factor promoting self-care neglect among nurses, negatively impacting their mental, physical, and spiritual well-being.^12^ Moreover, factors contributing to this population’s fragmented self-care are linked to the reality that professional nurses often have more than one job and work overtime, which leaves them physically drained.^7^

Nursing is a highly challenging profession impacting professional nurses’ physical and mental well-being. This population faces the demands of primary health care patients and a shortage of human resources, exacerbating their tiredness, anxiety, frustration, and exhaustion.^13,14^ In support of these findings, a study in Ghana found that inadequate motivation, inadequate staffing norms, managing many patients alone, and a lack of tea and lunch breaks contribute to professional nurses’ stress, burnout, and inability to practise self-care.^15^ Ultimately, professional nurses who are motivated and find themselves in a well-resourced work environment with managers who provide support are more likely to engage in self-care practices.^16^ Organisations should thus develop internal plans and policies that encourage an environment that accelerates self-care practices among professional nurses in primary health care settings.^17^ This study aimed to explore and describe professional nurses’ experiences of self-care practices in primary health care clinics in Ekurhuleni, Gauteng.

## Research methods and design

### Study design

A qualitative descriptive phenomenological research method was used for this study. This approach presents a holistic description of everyday conscious experiences, allowing researchers to set aside their preconceived ideas or opinions.^18^ Phenomenological studies explore the human experience using descriptions presented by the people involved.^19^ A phenomenological approach also provides a deeper meaning of participants’ experiences of the phenomenon of interest.^18^ The primary author used a phenomenological method to explore professional nurses’ experiences with self-care practices to understand the phenomenon from their point of view.

### Setting

This study was conducted in the City of Ekurhuleni (Ekurhuleni is a Xitsonga word, meaning ‘place of peace’), one of Gauteng’s five districts and one of the eight metropolitan municipalities of South Africa. This sub-district includes Brakpan, Springs, Daveyton, Geluksdal, Nigel, and Duduza. The sub-district has 25 primary health care clinics, three community healthcare centres, and two provincial hospitals. IsiZulu is the most spoken native language in the region. The setting of this study was in 10 primary health care clinics in the eastern sub-district where curative, and chronic health services are provided at no cost to the community.

### Population and sampling strategy

The population comprised 269 professional nurses employed in primary health care clinics in the eastern sub-district of Ekurhuleni. Non-probability, purposive sampling was used, and 20 professional nurses were interested in participating. Ten professional nurses from different primary health care facilities consented and participated in this study. The other 10 did not meet the inclusion criteria, did not honour the scheduled interviews, or decided not to partake after the information session. The inclusion criteria stipulated that participants must be experienced professional nurses employed for at least 1 year (12 months) in primary health care clinics, willing to share their knowledge, and sign an informed consent form. Participants were recruited through the distribution of flyers, which contained information about the study and the primary author’s telephone number and email address. Moreover, the research details were shared with the senior primary health care manager and other middle managers to distribute the information to the various professional nurses in primary health care facilities.

### Data-collection methods

Unstructured interviews are helpful as they serve as open communication with a purpose, where the researcher gathers more information about the participants’ world.^20^ In this study, unstructured interviews were conducted in English and recorded verbatim. The primary author posed one central question: ‘*What is your experience with self-care practices*?’ This question allowed the professional nurses employed within a primary health care clinic to describe their perspectives. It also allowed the authors to draw meaning and understanding of the described phenomenon.

One pilot interview was facilitated by the primary author and conducted through Microsoft (MS) Teams as per the participant’s request. During the interview, the primary author used different communication skills and reflective journals and made comprehensive field, observational, theoretical, and personal notes. The participant had access to free data, a compatible computer, and a room that ensured privacy. Moreover, the second author, who acted in a supervisory role, also logged on to listen to the interview. The pilot interview lasted approximately 45 min, which allowed the authors to assess whether the interview process was feasible. Thereafter, nine face-to-face interviews, which lasted approximately 45 min – 60 min, were conducted between 02 December 2020 and 08 March 2021. The pilot interviews were included in the data analysis process.

### Data analysis

Colaizzi’s descriptive phenomenological seven-step process was used to analyse the data^21^:

- Step 1: All audio recordings were transcribed verbatim, and the authors read the transcripts repeatedly to gain a clear understanding of participants’ thoughts, feelings, and views about the phenomenon.
- Step 2: The primary author carefully analysed all the transcripts to identify critical statements.
- Step 3: The primary author attached a general meaning to each statement from the verbatim quotes.
- Step 4: The interpretive meaning of events and situations was presented and arranged into themes and sub-themes.
- Step 5: Steps one and two were integrated into a complete description of professional nurses’ experiences of self-care practices.
- Step 6: The primary author presented and discussed the possible themes and sub-themes with a qualified independent coder. Moreover, the authors and independent coder validated the results and reached conclusions after separate, tentative coding was conducted. Additionally, a mutual consensus was reached between the authors and the independent coder on the study’s final themes and categories.
- Step 7: The primary author sought to verify the findings’ fundamental structure. In this study, the primary author presented the final themes to participants to determine if their experiences were accurately represented; this was done during a one-on-one consultation with the participants, and no changes were required to the themes and sub-themes.

### Trustworthiness

The principles of trustworthiness were upheld throughout the research.^22^ The authors promoted credibility through prolonged engagement. At the end of the data collection and analysis process, the primary author also confirmed with participants whether the themes reflected what they intended to say during the interviews. Confirmability and dependability were assured by both authors being involved in the data analysis and coding process, and an experienced independent coder offered a final analysis of the themes and categories.^22^ Transferability was attained by employing purposive sampling and providing a dense description of the participants’ demographics and the context in which this study was conducted.

### Ethical considerations

All participants attended individual information sessions, and the primary author emphasised voluntary participation. Participants were also informed that they could withdraw from the study without any punishment. Moreover, the authors upheld all ethical principles by adhering to autonomy, beneficence, non-maleficence, and justice. The primary author also approached a multidisciplinary team of psychologists to assist the participants if they experienced any emotional discomfort, and participants received these contact numbers to call at any time.

The University of Johannesburg, Faculty of Health Science, and the Research Ethics Committee, NHREC (Registration number: REC-504-2020) and the Higher Degrees Committee (HDC-01-22-2020) approved the study; certification was issued to the researchers. To enter the field to collect data, permission was also received without objection from the Ekurhuleni Health District Research Ethics Committee (NHRD No: GP-20200-032).

## Results

### Participants’ demographics

The participants’ demographics and characteristics are presented in Table 1.^23^ The participants’ ages ranged from 30 years to 51 years, their work experience ranged from 4 years to 25 years in a primary health care clinic, and all the participants had a degree or diploma in nursing (general nursing science, community, psychiatry, midwifery). Three professional nurses had a postgraduate diploma in clinical nursing science, health assessment treatment, and care (R48).

**TABLE 1:** Participants’ demographics.

Participant	Age	Gender	Employment in a primary health care facility (years)
Participant 1	34	Female	10
Participant 2	34	Male	4
Participant 3	45	Female	16
Participant 4	40	Male	8
Participant 5	51	Female	25
Participant 6	39	Male	7
Participant 7	49	Female	11
Participant 8	37	Female	14
Participant 9	30	Male	6
Participant 10	32	Male	7

*Source*: Nkabinde-Thamae GJ. Practices of self-care by professional nurses working at a primary health care clinic in Gauteng. University of Johannesburg, South Africa; 2021

Table 2 gives insight into the themes and sub-themes that were unveiled after data analysis. The overview of the themes and sub-themes was that factors inside and outside the participants’ world contributed towards them not to promote self-care practices. The inability of the participants to practise self-care appeared to have unfavourable consequences for them.

**TABLE 2:** Research findings – Themes and sub-themes.

Themes	Sub-Themes
1. Participants experienced internal and external factors that compromised self-care practices.	1.1. Inadequate resources (human resources such as nursing personnel, basic resources such as dressing packs, hand soap, gloves, masks, and sanitisers, personal protective equipment) contribute to self-care neglect. 1.2. Depressed economy: life is too expensive to maintain; the survival of the fittest resulting in the participants working overtime.
2. Holistic well-being is compromised by self-care neglect.	2.1. Participants experienced emotional distress such as sadness, stress, depression, feeling drained, and high levels of anxiety, irritability, and frustration.

*Source*: Nkabinde-Thamae GJ. Practices of self-care by professional nurses working at a primary health care clinic in Gauteng. University of Johannesburg, South Africa; 2021

### Theme 1: Participants experienced internal and external factors that compromised self-care practices

#### Sub-theme 1.1: Inadequate resources contribute to self-care neglect

The participants expressed that insufficient human and non-human resources resulted in their inability to practise self-care. However, despite the depleted resources that made it impossible to effectively execute their functions as professional nurses, they were still eager to remain in clinical practice and provide patient care. It appeared that the work environment was not promoting professional nurses’ self-care practices:

‘There is simply no time to practice self-care; as nurses, we are overworked, there is a significant shortage of nurses in practice … We all see more patients than standard, in terms of norms and standards, but what can we do or say? We took the pledge, and the pace of work is also so fast.’ (Participant 10, 32 years old, Male)

Professional nurses employed at primary health care clinics find it difficult to practise self-care. They face various obstacles, such as unconscious self-neglect, the demands of the nursing profession, and prioritising the needs of others:

‘So it is demanding where it comes to a point where you neglect your own needs, it is demanding to an end even if you are sick, you sick but you still say if I do not go to work, already we a limited number of professional nurses at work, if I do not go there will be more strain on my colleagues, I will instead go with my flue to work, self-treat myself and take it from there, and only to find out in the long run it complicates, because you never went to the doctor, never had time to consult and proper test done, so the demands go as far as that and to be honest no care not even management. However, what can one do? I love my patients and profession, being aware of the shortage of professional nurses. In general, I feel overwhelmed when resources are not sufficient.’ (Participant 1, 34 years old, Female)

‘The shortages include work overload, resources, and equipment that we use, Uhhhh Personal Protective Equipment. I think that is the main contributory factor. Yes, support from management contributes to one feeling emotionally exhausted, resulting in having no energy to engage in self-care practices.’ (Participant 5, 51 years old, Female)

Another participant unveiled the extent to which shortage of personal protective equipment (PPE) contributed to self-care neglect:

‘Personal protective equipment is supposed to be available in primary health care facilities, but our clinics don’t have the basics, like gloves, aprons and hand sanitizers, the operational managers indicate there is not enough budget to procure those personnel protective equipment.’ (Participant 5, 51 years old, Female)

‘The insufficient personal protective equipment stresses me, and the situation is worse now because, its COVID, I worry will contract the disease, and give it to my family, one works without applying personal protective equipment, because I not caring for myself, so that is not self-care.’ (Participant 10, 32 years old, Male)

The participants shared that they often cannot engage in self-care practices as they are exhausted and have no energy or desire to do so:

‘In 2019 and late 2020, I was exhausted and emotionally drained because in the facility I am working in, two nurses were on long sick leave, and a couple of nurses were absent, or on annual leave, so the remaining nurses had to do double services, I struggle to practice self-care even more during that period.’ (Participant 7, 49 years old, Female)

These verbatim quotes illustrate the challenges professional nurses experienced within the nursing profession, prioritising the needs of others, and neglecting their health even if they were aware of the possible consequences. Staff shortages were the most significant challenge impeding professional nurses’ self-care practices, often leaving them physically and emotionally depleted. Moreover, it appears the participants were suffering from compassion fatigue, and there was a clear outcry for support from management.

Compassion fatigue appeared to be linked with self-care neglect in the world of these participants:

‘I am in a profession that requires one to be compassionate and show empathy towards others daily and provide extraordinary care irrespective of what you might be experiencing on a personal level, one’s health is compromised, and there is nothing you can do to uphold or improve your health or wellness. I am in a profession that desires me to give more or all of me until there is nothing left of me; I am exhausted, and being in service for ten years, is still early days, but I am already exhausted. I assume it is because of the dedication I and others have and my profession. As a result, one gives into compassion fatigue without noticing.’ (Participant 4, 40 years old, Male)

Moreover, participants made a desperate plea for support from their managers:

‘My recent experiences with uncaring moments or experiences in the nursing profession is the lack or no support from managers. You do what your managers expect of you, working even at times beyond the scope of duty. Thus, you neglect yourself, and managers do not support or refer you for help when they see you on the verge of losing your sanity.’ (Participant 9, 30 years old, Male)

‘Honestly speaking, I sometimes feel that managers are not on our side as most expect us to push queues and compromise ourselves, as longest the work is done, then all is well.’ (Participant 8, 37 years old, Female)

Professional nurses seemed compelled to do what was expected of them, even beyond the call of duty. As a result, they experienced burnout syndrome.

#### Sub-theme 1.2: Depressed economy: Life is too expensive to maintain; the survival of the fittest

The participants deemed self-care unattainable; they used their time for self-care to work overtime and sustain their families. They shared that they felt overwhelmed and financially over-committed. The state of the economy was affecting them, as many people lost their jobs and relied on the participants to provide for them financially. Some participants were also single parents with overwhelming responsibilities:

‘I don’t have a personal life because I am always at work; I also work for a locum to do extra cash because even my family depends on me. We are underpaid; that’s why I am working, Uhhhh locum because my family, like my patients, depend on us to provide quality care.’ (Participant 3, 45 years old, Female)

‘If I reflect, I don’t have time in general for myself, I am always exhausted, I work a 40-hour shift, and remember, I work in an environment where there is a shortage of nurses, so I don’t just do my work, and on weekends, I work overtime.’ (Participant 2, 34 years old, Male)

Participants’ reflections offered insight into why professional nurses work overtime, supplementing their current salaries to sustain their lifestyles and help those around them. However, during the data-collection process, it became evident that overtime work also has negative consequences. The following participants shared the impact thereof:

‘I feel sad because I always feel exhausted, and because I am working for locum, I even forget to take my medication correctly, and I do not have time for my family, and I cannot even cook for my children because I am always not available at home.’ (Participant 5, 51 years old, Female)

‘Everyone could see I was tired or on the verge of losing my mind, but I was still expected to be Ok and still give and be present to ensure nurses are not forgotten and prioritized; nurses are also important; we should be allowed to practice self-care.’ (Participant 7, 49 years old, Female)

Participants’ real-life experiences thus highlight the negative consequences of working overtime, particularly on their mental health and well-being.

### Theme 2: Holistic well-being is compromised by self-care neglect

#### Sub-theme 2.1: Participants experienced emotional distress such as sadness, stress, depression, feeling drained, high anxiety levels, irritability, and frustration, affecting their holistic well-being

The participants reported high levels of emotional distress, as they felt they were not coping with the nursing profession and their personal lives. These feelings prevented them from engaging in self-care practices, and they felt their mental well-being was compromised.

Participant 9 explicitly shared how emotional stress stripped him of the opportunity to practise self-care:

‘So being psychologically and mentally overwhelmed is preventing one from practising self-care.’ (Participant 9, 30 years old, Male)

Other participants mentioned the impact that intense, excessive, and persistent worry had on them:

‘Anxiety is an uncomfortable feeling, and it feels as if you are weak. Do you know I never suffered from anxiety? However, as one experiences all these challenges in nursing, one cannot help one feel anxious. So, I must say anxiety is paralyzing me, in return, I cannot practice self-care, because you feel fatigued and having palpitations … sometimes it is so bad it feels as if you are trembling.’ (Participant 10, 32 years old, Male)

‘Your mental health is compromised you end up not being able to take care of your mental state because in your mind, you think that I am selfless, stressed, and overthinking; I am trying to help the next person, while your mental health is suffering, your body is suffering, your emotions are suffering, while you are unaware … you are unaware.’ (Participant 2, 34 years, Male)

‘Stress is a terrible thing … sometimes you feel that suicide is the only way out … because feeling stressed and on the verge of suicide is not lovely; I am on some anti-depressants. No one knows I hide it very well, but I am sad, and I think some of my colleagues can see I am stressed and miserable.’ (Participant 6, 39 years old, Male)

The challenges participants experienced affected their mental health, compromising their holistic well-being. These intense feelings and emotions had undesired effects on the participants’ lives. Moreover, professional nurses who did not practise self-care in totality reported that their lives spiralled out of control. Participants were ultimately unable to seek assistance as they wanted to avoid the stigma attached to mental illness.

## Discussion

The authors gained insight into professional nurses’ lived experiences with self-care practices. This discovery promotes awareness of the contributory factors that impede professional nurses’ self-care. The findings reflected the fact that the lack of human resources significantly affects professional nurses’ ability to practise self-care. Most healthcare facilities in South Africa are experiencing a shortage of essential resources, such as dressing packs, hand soap, gloves, masks, and sanitisers, which compromise professional nurses’ well-being as they are constantly overwhelmed and provide care in fear.^24^ The lack of available PPE risks professional nurses’ health, leaving them more vulnerable in nursing.^25^ A recent study in a primary health care environment unveiled the extent to which professional nurses and nurse managers stretched themselves during the COVID-19 pandemic and how limited resources in primary health care facilities contributed to emotional turmoil among healthcare workers.^26^ However, it has also been found that when sufficient PPE is available, professional nurses still choose not to wear it effectively. They fear it may not be available when needed, leading to unproductivity and non-compliance, risking their health.^27^ The absence of PPE within a healthcare organisation also leads to constant anger, inhospitality, and self-care neglect among workers.^28^ Ultimately, it is a legal obligation for health organisations to ensure the availability of PPE to prevent moral and ethical distress among nurses.^29^

The study also revealed that professional nurses experience decreased empathy and sympathy because of insufficient resources, compromising ethical decisions within the nursing profession and further promoting emotional distress and possible negligence.^30^ However, despite the resource challenges within the healthcare facilities and the general impact on professional nurses’ well-being, nurses remain in practice providing care.

Participants reflected an evident yearning for support from management. A supportive manager is a person who provides mentoring, gives feedback, resolves problems, and creates an enabling environment where individuals can practise self-care through adequate resource allocation.^31^ Similar findings were reported in a study conducted in a midwifery context, indicating midwives worked in unfavourable conditions, which led to unproductivity as there was no support from management when needed.^32^ Support from management is essential in the workplace and assists in advocating for nurses who need clarity about specific issues when they are emotionally overwhelmed.^33^

Within this study, some internal factors compelled the participants not to practise self-care, such as working overtime, which deprived them of sleep. The number of nurses working overtime has increased globally because of a staff shortage but also for their financial gain, though it negatively affects their health.^34^ In the South African context, it was observed that professional nurses’ overtime shifts in all healthcare settings increased during the pandemic to care for patients, even without getting paid or receiving any form of acknowledgement.^35^ However, despite the rationale for professional nurses to work overtime, the extent of that overtime had a ripple effect thus hindering their ability to engage in self-care practices.^36^ A study conducted during the COVID-19 pandemic revealed nurses worked 12-h shifts or longer because of staff shortages. These nurses performed poorly because of physiological strain, fatigue, burnout, and job dissatisfaction. Overtime also contributes to poor physical health and injuries, increased alcohol consumption, physical inactivity, and depression among nurses.^37^ If nurses work overtime to the extent that they are physically and mentally exhausted, it will not only hurt them but put the lives of their patients at risk.^38^

Professional nurses know they must care for themselves but struggle to incorporate self-care practices into their daily lives; they must learn to dedicate time to their well-being.^39^ In this study, the authors noted that most professional nurses have more than one job contributing to their monthly income, preventing them from having time for themselves. This may be perceived as a lack of priority towards themselves, as they constantly experience anxiety, agitation, and exhaustion. Caring for the self will help individuals engage positively with those around them and their environment.^40^ It was also seen that the COVID-19 pandemic largely contributed to nurses’ mental health problems as they worked overtime and faced a shortage of PPE.^41,42^ However, the main contributory factors to professional nurses’ emotional distress within the profession are uncertainty, extremely stressful clinical situations, and conflict with patients, families, and other care providers.^43,44^ Evidence shows that most nurses experience anxiety and depression, potentially leading to compromised self-care practices and poor patient management.^45^ Long working hours and an inability to spend adequate time with loved ones predispose nurses to mental health problems.^46^ In addition, stress and burnout are almost impossible to avoid in the nursing profession because of the challenges and demands of the work; the only remedy would be to provide strategies that may assist professional nurses in dealing with these challenges.^47^ The stress and burnout syndrome deprive the world of experienced nurses, preventing them from practising self-care and affecting their work performance and general well-being.^48^ There also appears to be a link between burnout and occupational stressors, which, if not prevented or minimised, will hurt nurses’ performances, contribute to absenteeism, and further decrease patients’ satisfaction.^15^

During this study, the authors became aware of professional nurses’ experiences with self-care practices, and participants did not have a voice to express what assistance they needed to practise self-care. Moreover, participants remained in practice even when they felt they could no longer bear the challenges within the profession; their unconditional love for nursing was evident, resulting in compassion fatigue. Researchers demonstrated that compassion is a fundamental principle in nursing, promoting and enhancing high-quality care.^49^ However, professional nurses are constantly exposed to patients and family members who feel helpless, hopeless, and dependent on them, which ultimately leads to compassion fatigue.^50,51^ Additional factors such as excessive workloads, a compromised work environment, extensive physical exhaustion, and emotional and spiritual meltdowns significantly contribute to compassion fatigue among this population.^52^

### Limitations of the study

Within this study, the factors that appeared to hinder professional nurses’ ability to practise self-care were limited to the experiences of 10 participants. The authors are mindful that other aspects might have contributed to the nurses not being able to practise self-care. Lastly, this study excluded other health professionals and was limited to a primary health care environment.

### Recommendations

Based on the internal and external factors that hinder self-care, interventions to sustain and maintain self-care practices will require a task-shifting approach. Various stakeholders should actively engage to assist professional nurses in making self-care practices possible. The South African Nursing Council should make it mandatory for healthcare institutions to include self-care practices and a focus on general well-being as part of their routine in-service training. Further research should be conducted to explore and describe other healthcare workers’ experiences with self-care techniques. In addition, hierarchy-level management (operational management, senior manager, and executive management) should endorse a positive practice environment to promote self-care.

## Conclusion

The research participants provided evidence that they struggled to practise self-care in their personal and professional lives. The collected data demonstrated various factors that hinder their self-care and how those should be mitigated. It is imperative to promote awareness of the need for self-care practices in the world of professional nurses.
